# TFEB-mediated increase in peripheral lysosomes regulates store-operated calcium entry

**DOI:** 10.1038/srep40797

**Published:** 2017-01-13

**Authors:** Luigi Sbano, Massimo Bonora, Saverio Marchi, Federica Baldassari, Diego L. Medina, Andrea Ballabio, Carlotta Giorgi, Paolo Pinton

**Affiliations:** 1 Dept. of Morphology, Surgery and Experimental Medicine, Section of Pathology, Oncology and Experimental Biology, Laboratory for Technologies of Advanced Therapies (LTTA), University of Ferrara, Ferrara, 44121, Italy; 2 Telethon Institute of Genetics and Medicine (TIGEM), 80078 Pozzuoli, Naples, Italy; 3 Dept. of Molecular and Human Genetics, Baylor College of Medicine, Houston, Texas 77030, USA; 4 Jan and Dan Duncan Neurological Research Institute, Texas Children’s Hospital, Houston, Texas 77030, USA; 5 Medical Genetics, Dept. of Translational Medicine, Federico II University, 80131 Naples, Italy

## Abstract

Lysosomes are membrane-bound organelles mainly involved in catabolic processes. In addition, lysosomes can expel their contents outside of the cell via lysosomal exocytosis. Some of the key steps involved in these important cellular processes, such as vesicular fusion and trafficking, require calcium (Ca^2+^) signaling. Recent data show that lysosomal functions are transcriptionally regulated by transcription factor EB (TFEB) through the induction of genes involved in lysosomal biogenesis and exocytosis. Given these observations, we investigated the roles of TFEB and lysosomes in intracellular Ca^2+^ homeostasis. We studied the effect of transient modulation of TFEB expression in HeLa cells by measuring the cytosolic Ca^2+^ response after capacitative Ca^2+^ entry activation and Ca^2+^ dynamics in the endoplasmic reticulum (ER) and directly in lysosomes. Our observations show that transient TFEB overexpression significantly reduces cytosolic Ca^2+^ levels under a capacitative influx model and ER re-uptake of calcium, increasing the lysosomal Ca^2+^ buffering capacity. Moreover, lysosomal destruction or damage abolishes these TFEB-dependent effects in both the cytosol and ER. These results suggest a possible Ca^2+^ buffering role for lysosomes and shed new light on lysosomal functions during intracellular Ca^2+^ homeostasis.

Over the past two decades, our understanding of how extracellular signals are conveyed to eukaryotic cells by increasing the intracellular Ca^2+^ concentration has improved. It is now common knowledge that a variety of extracellular stimuli ranging from the binding of hormones, neurotransmitters, and growth factors to phenomena such as cell-cell interactions occur through diverse mechanisms (e.g., receptors that are themselves ion channels, have intrinsic enzymatic activity, or are coupled to enzymatic effectors via G proteins) to induce increases in cytoplasmic Ca^2+^ concentrations ([Ca^2+^]_c_) that exhibit defined amplitudes and kinetics^1^,^2^,^3^.

In eukaryotic cells, a large electrochemical Ca^2+^ gradient exists across the plasma membrane (PM) (approximately 70 to 90 mV), but the [Ca^2+^]_c_ is less than 1/10,000 that of the extracellular milieu. However, eukaryotic cells can store Ca^2+^ in many organelles and can mobilize the ion in response to endogenous and extracellular stimuli. The major intracellular Ca^2+^ storage unit is the ER (luminal [Ca^2+^]_ER_ 500 μM-1 mM)^3^, which exhibits significant heterogeneity in the Ca^2+^ level among its sub-regions. Upon stimulation with agonists such as histamine or ATP, the ER rapidly releases Ca^2+^ through the inositol 1,4,5-trisphosphate receptor (IP3R), thereby generating transient waves in the cytoplasm and mitochondria to promote cell activities^4^,^5^. Upon ER Ca^2+^ depletion, the luminal sensor protein STIM1 oligomerizes on the ER membrane and migrates to sites of ER/PM interaction to activate the highly Ca^2+^-selective ORAI channels located on the PM^6^,^7^. Thus, the ER Ca^2+^ store is replenished via the sarco-/endoplasmic reticulum Ca^2+^-ATPase (SERCA) pump^8^,^9^,^10^,^11^ in a process known as capacitative Ca^2+^ entry or store-operated Ca^2+^ entry (SOCE). Similar to the ER^8^,^10^, lysosomes act as intracellular Ca^2+^ stores with a free Ca^2+^ concentration of ~0.4–0.6 mM^12^,^13^, which is 3–4 orders of magnitude higher than the cytosolic Ca^2+^ concentration (~100 nM). Although the depletion of lysosomal Ca^2+^ stores does not induce extracellular Ca^2+^ entry via SOCE, capacitative Ca^2+^ entry induced by ER Ca^2+^ release might contribute to the accumulation of Ca^2+^ inside lysosomes^14^. A role for Ca^2+^ in lysosomal function is supported by the well-established paradigm of its role in organelle and PM fusion^15^, and lysosomes have only recently been considered an intracellular Ca^2+^ signaling center^16^. In particular, Ca^2+^ release from the lysosome has been shown to be required for late endosome-lysosome fusion^17^, lysosomal exocytosis, phagocytosis, membrane repair, signal transduction^9^,^18^,^19^, and the induction and modulation of the autophagic pathway^20^. Furthermore, the characterization of lysosomal Ca^2+^-releasing factors such as NAADP^21^ or ML-SA1^22^ has provided evidence of Ca^2+^-dependent functional coupling between the ER and lysosomes^21^.

The principal Ca^2+^ channel in the lysosome, Mucolipin 1 or TRP channel 1 (MCOLN1 or TRPML1), as well as lysosomal Ca^2+^ sensors such as the C2 domain-containing synaptotagmin VII, are also required for many of these functions^9^,^19^,^23^. In contrast, a reduction in the lysosomal Ca^2+^ content caused by mutations of the human TRPML1 gene is considered to be the primary pathogenic cause underlying some lysosomal storage diseases and common neurodegenerative diseases^13^,^24^, such as type IV Mucolipidosis^25^.

The identification of transcription factor EB (TFEB)^26^,^27^ as a master regulator of lysosome function has revealed how the lysosome adapts to environmental cues. In particular, TFEB was observed to bind a palindromic 10-base pair motif 26 (5′-GTCACGTGAC-3′) that is highly enriched in the promoter region of the 96 identified lysosomal genes^28^, thereby inducing their expression, which leads to increased lysosomal biogenesis as well as exocytosis and fusion of the lysosome with the PM and to lipid catabolism^27^. Interestingly, TFEB-mediated regulation of lysosomal exocytosis plays an important role in osteoclast differentiation and bone resorption^29^. Furthermore, TFEB, similar to many other transcription factors, undergoes a complex sequence of phosphorylation and dephosphorylation. The TFEB protein can be phosphorylated in the cytosol via several distinct molecular pathways: i) extracellular signal-regulated kinase (ERK2)^30^,^31^, ii) mechanistic target of rapamycin complex 1 (mTORC1)^31^,^32^,^33^ and iii) protein kinase Cβ (PKCβ)^29^. Interestingly, cytoplasmic TFEB is located both in the cytosol and on the lysosomal surface, where it interacts with mTORC1 and lysosome nutrient sensing (LYNUS) machinery as a component of nutrient sensing^32^. Recently, a new lysosomal signaling pathway activated by lysosomal calcium release through TRPML1 has been shown to be involved in the activation of TFEB via de-phosphorylation^20^.

During lysosomal exocytosis, TFEB induces both the docking and fusion of lysosomes with the PM in a process that requires the lysosomal calcium channel TRPML1^34^.

Based on these observations, we investigated the contribution of TFEB expression and lysosomes to intracellular Ca^2+^ homeostasis with the goal of characterizing Ca^2+^ dynamics in both the cytosol and ER upon the induction of SOCE.

## Results

### TFEB overexpression promotes lysosomal localization to the PM to decrease capacitative Ca^2+^ influx

To investigate the role of TFEB and lysosomes in intracellular Ca^2+^ homeostasis, we first evaluated the effects manipulating its expression. We transiently overexpressed TFEB (TFEB-3xflag) for 48 h in human cervical carcinoma (HeLa) cells (Supplementary Fig. S1A), revealing an increase in protein expression compared with control cells transfected with pcDNA3.

As previously demonstrated by Sardiello *et al*.^26^, the primary consequence of this modified genetic control is increased lysosomal biogenesis due to the upregulation of most lysosomal proteins modulated by the CLEAR consensus sequence in promoter regions^27^. To verify these data and validate our experimental setup, we analyzed lysosomal morphology using 3D imaging deconvolution. The lysosomal compartment was marked by the overexpression of Lysosomal-associated membrane protein 1 associated with green fluorescence protein (LAMP1-GFP). Imaging analysis revealed a significant expansion of the lysosomal compartment (see Supplementary Fig. S1B) in HeLa cells overexpressing TFEB (433.22 ± 17.11 objects in control cells vs. 528.32 ± 30.18 objects in TFEB-overexpressing cells).

**Figure 1:**
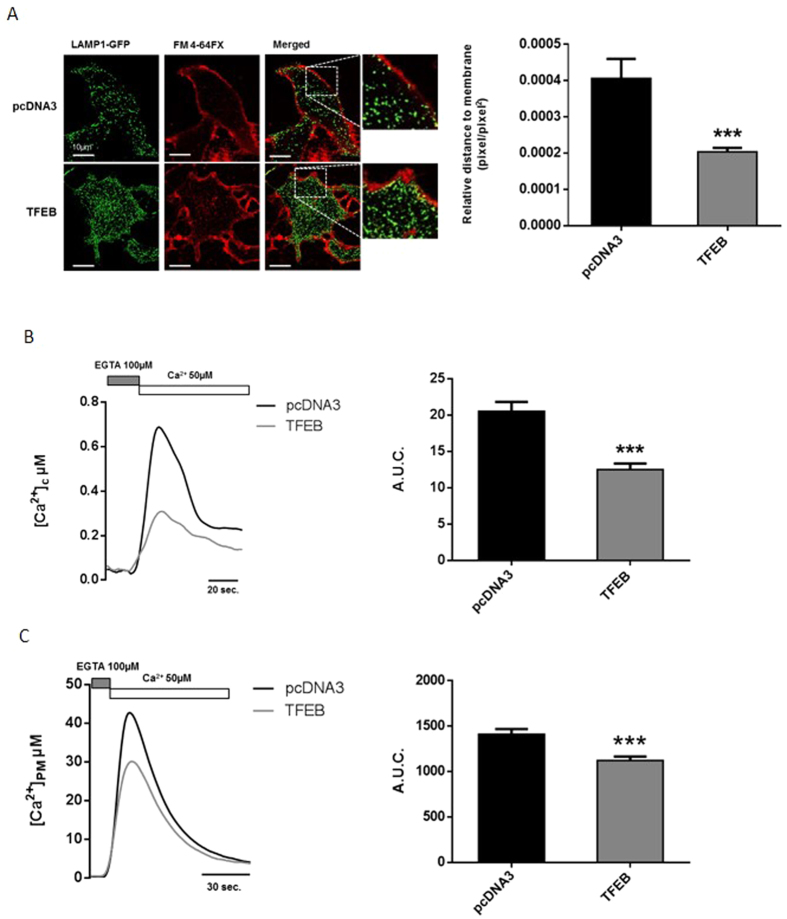
TFEB overexpression increases lysosomal localization to the plasma membrane, decreasing capacitative Ca^2+^ entry. (**A**) Representative images and quantification of the distance of lysosomes from the plasma membrane, normalized to the cellular area, in HeLa cells co-transfected with pcDNA3 (control) or TFEB3xflag (TFEB) and LAMP1-GFP (green) for 48 h; the plasma membrane is stained with the FM4-64fx colorant (red) (pcDNA3 n = 12, TFEB n = 15). (**B**) Capacitative Ca^2+^ entry was measured using cytosolic aequorin in control HeLa cells (pcDNA3) or HeLa cells overexpressing TFEB for 48 h after Ca^2+^ (CaCl_2_ 50 μM) administration (pcDNA3 n = 17, TFEB n = 20). (**C**) Capacitative Ca^2+^ entry was measured using PM-targeted aequorin in control HeLa cells (pcDNA3) or HeLa cells overexpressing TFEB after Ca^2+^ (50 μM) administration (n = 10 for each condition). Data are presented as the means ± SEM, ***p < 0.001. a.u.c. = area under the curve.

It has been suggested that TFEB overexpression increases the pool of lysosomes proximal to the PM and promotes their fusion with the PM^34^. To confirm these observations, we performed experiments aimed at assessing the location of the lysosomes (see Methods). We analyzed the distance of LAMP1-GFP-marked lysosomes from the FM4-64fx dye-stained PM (see Fig. 1A), which revealed that these organelles are proximal to the PM (Fig. 1A) in HeLa cells transfected with TFEB-3xFLAG compared with the control (4.07E^−04^ ± 4.13E^−05^ pixel/pixel^2^ for control vs. 2.04E^−04^ ± 2.14E^−05^ pixel/pixel^2^ for TFEB).

Next, we considered the possibility that an increased number of lysosomes and their proximity to the PM might contribute to intracellular Ca^2+^ homeostasis, particularly for phenomena occurring near the PM. We took advantage of aequorin technology^35^ to assess the level of accumulated cytosolic Ca^2+^ entering the cell across the PM via SOCE in the cell models previously described. To induce capacitative Ca^2+^ influx via the activation of SOCE, HeLa cells were pre-treated with a SERCA blocker (thapsigargin; 200 nM) for 15 min in a Ca^2+^ free medium (KRB/EGTA) to encourage the depletion of ER Ca^2+^ stores. When the release of stored ER Ca^2+^ was complete, Ca^2+^ was added to the KRB perfusion. A major increase in [Ca^2+^]_c_ due to Ca^2+^ influx through plasma membrane channels was observed when the perfusing buffer was supplemented with 1 mM CaCl_2_. Under these conditions, TFEB overexpression did not significantly influence SOCE (cytosolic Ca^2+^ uptake in control HeLa cells = 114.53 ± 3.97 a.u.c. vs. TFEB-overexpressing cells = 111.47 ± 3.66 a.u.c.) (Supplementary Fig. S2). However, because of the influx of massive amounts of Ca^2+^ into the subplasmalemmal region (not physiological, but due to the Ca^2+^-free pre-treatment) and the discharge of the aequorin probe due to the high Ca^2+^ affinity that has been ascribed to lysosomes^12^, the [Ca^2+^]_c_ values obtained under these conditions were largely artifactual, as reported previously^36^. To avoid saturation of the aequorin probe in subsequent experiments, cells were therefore challenged with a lower extracellular [Ca^2+^] of 50 μM.

In our experimental setting, this procedure evoked an increase in the [Ca^2+^]_c_ followed by a gradual decline, providing evidence for the presence of an abundance of SOCE in HeLa cells (Fig. 1B).

Samples transfected with the TFEB construct displayed a consistent and significant reduction of cytosolic Ca^2+^ accumulation after the activation of capacitative Ca^2+^ influx compared with the control samples (cytosolic Ca^2+^ uptake in control HeLa cells = 20.57 ± 1.30 a.u.c. vs. TFEB-overexpressing cells = 12.54 ± 0.84 a.u.c.).

To confirm that the presence of lysosomes in the proximity of the PM might modulate SOCE, we measured the Ca^2+^ influx using a different aequorin probe targeting the cytosolic side of the PM (PM-Aeq)^36^. After the activation of SOCE via depletion of ER Ca^2+^ stores, Ca^2+^ (CaCl_2_ 50 μM) was added to the KRB perfusion (Fig. 1C). In our experimental setting, HeLa cells transfected with the TFEB construct displayed a consistent and significant reduction of Ca^2+^ accumulation in close proximity to the PM compared with the control cells (PM Ca^2+^ uptake in control HeLa cells = 1410.30 ± 59.05 a.u.c. vs. TFEB-overexpressing cells = 1122.78 ± 42.22 a.u.c.).

Overall, these data suggest a specific role for TFEB in regulating SOCE, likely due to increased lysosomal localization at the PM.

### Impairment of lysosomal activity restores normal capacitative Ca^2+^ influx in TFEB-expressing cells, and TFEB knockdown increases Ca^2+^ influx via SOCE

To determine whether the TFEB-dependent effects on SOCE are strictly related to altered lysosomal activity, we used different pharmacological approaches that compromise lysosomal structure and function through different modes of action.

The first compound was the membrane-permeable di-peptide glycyl-L-phenylalanine 2-naphthylamide (GPN), which causes selective osmotic lysis of the lysosomal membrane triggered by cleavage of the lysosomal enzyme cathepsin C^37^. Its specific lysosome-perforating activity allows it to be used for mobilizing lysosome-specific Ca^2+^ stores^14^,^38^,^39^,^40^,^41^.

**Figure 2:**
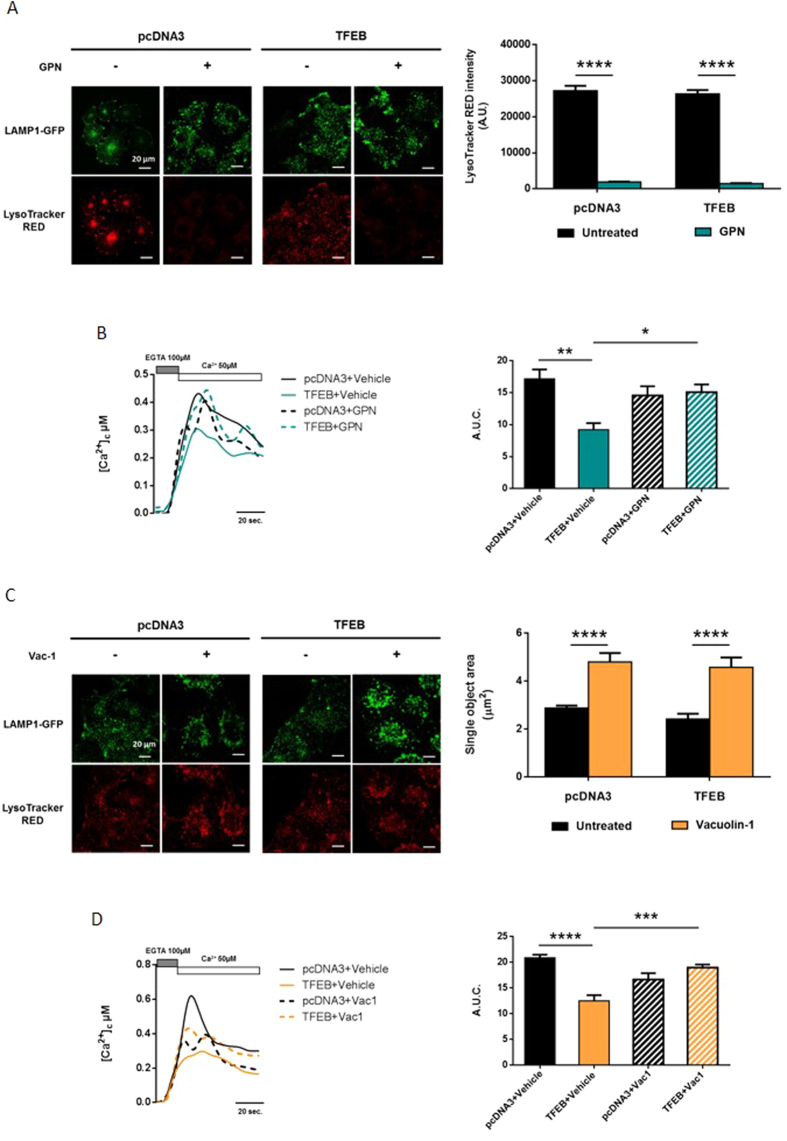
Impairment of lysosomal activity restores capacitative Ca^2+^ influx. (**A**) Representative images and quantification of the LysoTracker RED (red) dye intensity after treatment of HeLa cells with 200 nM thapsigargin for 30 min in the absence of Ca^2+^, with or without GPN 200 μM; the cells were transfected with LAMP1-GFP (green) to highlight lysosomes (pcDNA3 + Vehicle n = 25, TFEB + Vehicle n = 25, pcDNA3 + GPN n = 32, TFEB + GPN n = 31). (**B**) Cytosolic Ca^2+^ measurements after SOCE activation, with aequorin in control HeLa cells (pcDNA3) or HeLa cells overexpressing TFEB, pretreated with GPN 200 μM or DMSO (vehicle) for 30 min (pcDNA3 + Vehicle n = 8, TFEB + Vehicle n = 8. pcDNA3 + GPN n = 13, TFEB + GPN n = 11). (**C**) Representative images and quantification of the area of single lysosomes after treatment of HeLa cells with 10 μM Vac-1 or DMSO (vehicle) for 1 h, causing fusion of lysosomes (enlargeosomes); the cells were transfected with LAMP1-GFP (green) to highlight lysosomes and stained with LysoTracker RED (red) dye (n = 90 for each condition). (**D**) Cytosolic Ca^2+^ measurements after SOCE activation, using aequorin in control HeLa cells (pcDNA3) or HeLa cells overexpressing TFEB, pretreated with 10 μM Vac-1 or vehicle for 1 h (pcDNA3 + Vehicle n = 6, TFEB + Vehicle n = 12, pcDNA3 + Vac-1 n = 11, TFEB + Vac-1 n = 10). Data are presented as the means ± SEM; *p < 0.05, **p < 0.01, ***p < 0.001, ****p < 0.0001. a.u.c. = area under the curve, a.u. = arbitrary units.

Following treatment with GPN, LysoTracker RED staining was abolished in both pcDNA3-transfected cells (mean LysoTracker RED intensity in untreated pcDNA3 HeLa cells = 27264.18 ± 1364.86 a.u. vs. LysoTracker RED intensity in GPN-treated cells = 1924.26 ± 95.69 a.u.) and TFEB-overexpressing HeLa cells (mean LysoTracker RED intensity in untreated TFEB HeLa cells = 26359.23 ± 1056.64 a.u. vs. LysoTracker RED intensity in GPN-treated cells = 1472.63 ± 100.17 a.u.), without affecting the distribution of LAMP1-GFP (Fig. 2A), indicating permeabilization of the lysosomal membrane.

Activation of SOCE after pre-treatment with GPN (200 μM) did not affect cytosolic Ca^2+^ accumulation in HeLa cells compared with the untreated condition (cytosolic Ca^2+^ uptake in control HeLa cells with vehicle = 17.16 ± 1.47 a.u.c. vs. control cells with GPN = 14.58 ± 1.42 a.u.c.) (Fig. 2B). Conversely, GPN treatment abolished the alterations induced by TFEB overexpression, suggesting a possible buffering role for the lysosome (cytosolic Ca^2+^ uptake of TFEB-overexpressing HeLa cells treated with a vehicle = 9.21 ± 1.01 a.u.c. vs. TFEB cells with GPN = 15.09 ± 1.20 a.u.c.).

The second compound, vacuolin-1 (Vac-1), promotes the alteration of lysosomal morphology, resulting in the fusion of acidic organelles without affecting the pH of the lysosomal lumen^42^. Nevertheless, Vac-1 blocks lysosomal exocytosis induced by Ca^2+^ ionophores and by membrane wounding^42^. Although the mechanism of action is unknown, previous studies have revealed that Vac-1 does not affect lysosomal ion transport mechanisms and only changes the surface area available to lysosomes, thereby perturbing their interactions with other organelles^43^. Thus, in the presence of Vac-1, lysosomes cannot fuse with the PM^44^. To confirm these data, we treated HeLa cells transiently expressing LAMP1-GFP and stained with LysoTracker RED with Vac-1 (10 μM for 1 h). We observed an altered and hyper-fused lysosomal compartment (Fig. 2C) without an evident modification in lysosomal permeability to LysoTracker RED in cells transfected with either the empty vector (single object area of untreated pcDNA3 cells = 2.88 ± 0.10 μm^2^ vs. single object area of Vac-1-treated HeLa cells = 4.80 ± 0.37 μm^2^) or the TFEB construct (single object area of untreated TFEB cells = 2.42 ± 0.22 μm^2^ vs. single object area of Vac-1-treated HeLa cells = 4.58 ± 0.41 μm^2^).

Then, we performed capacitative Ca^2+^ influx experiments on HeLa cells transiently transfected with empty vector (pcDNA3) or TFEB after pretreatment with Vac-1 (10 μM for 1 h) or DMSO (vehicle). Under experimental conditions including only vehicle, we observed maintenance of the differences in capacitative Ca^2+^ influx between control and TFEB-overexpressing samples (Fig. 2D). In contrast, Vac-1 treatment abolished the TFEB-related effects on SOCE (cytosolic Ca^2+^ uptake of TFEB overexpressing HeLa with vehicle = 12.49 ± 1.10 a.u.c. vs. TFEB cells with Vac-1 = 18.96 ± 0.62 a.u.c., pcDNA3 cells with vehicle = 20.86 ± 0.60 a.u.c., pcDNA3 cells treated with Vac-1 = 16.66 ± 1.23 a.u.c.).

To determine whether the effects of altered lysosome activity on SOCE are mediated by TFEB-dependent alteration of the lysosomal compartment, we transiently inhibited the expression of the transcription factor in HeLa cells for 72 h (see Supplementary Fig. S3A). To validate our experimental setup, we analyzed lysosomal morphology using 3D imaging deconvolution. As expected, imaging revealed a significant reduction in the number of lysosomes (see Supplementary Fig. S3B) in TFEB-silenced cells (697.18 ± 27.50 objects in control siRNA HeLa cells vs. 567.09 ± 17.01 objects in TFEB siRNA cells). Furthermore, to consolidate our experimental model and confirm the role of TFEB as a key regulator of lysosomal localization to the PM, we analyzed the intracellular distribution of lysosomal compartments (as previously described) by measuring their distance from the PM (see Supplementary Fig. S3C). Compared to the control, we observed that lysosomes were located farther from the PM (Supplementary Fig. S3C) in TFEB-depleted HeLa cells (7.47^−04^ ± 3.14E^−05^ pixel/pixel^2^ for control siRNA cells vs. 8.67E^−04^ ± 2.23E^−05^ pixel/pixel^2^ for TFEB siRNA HeLa cells).

Subsequently, we assessed the level of cytosolic Ca^2+^ entering the cell across the PM via SOCE employing the same experimental setting used for TFEB-overexpressing HeLa cells. After induction of capacitative Ca^2+^ influx, Ca^2+^ (CaCl_2_ 50 μM) was added to the KRB perfusion, and we observed that TFEB-silenced cells (see Supplementary Fig. S3D) displayed a significant increase in cytosolic Ca^2+^ uptake compared with the control samples (cytosolic Ca^2+^ uptake in control siRNA HeLa cells = 17.63 ± 0.78 a.u.c. vs. TFEB siRNA cells = 25.68 ± 1.87 a.u.c.). Overall, these data suggest that the effect of TFEB activity on SOCE is strictly dependent on both lysosomal activity and the PM-specific positioning of lysosomes.

### TFEB reduces SOCE-dependent Ca^2+^ refilling in the ER

As seen above, the capacitative influx is activated in response to the depletion of intracellular Ca^2+^ in the ER, and it represents the main path for the maintenance of Ca^2+^ levels in the ER during stimulation.

**Figure 3:**
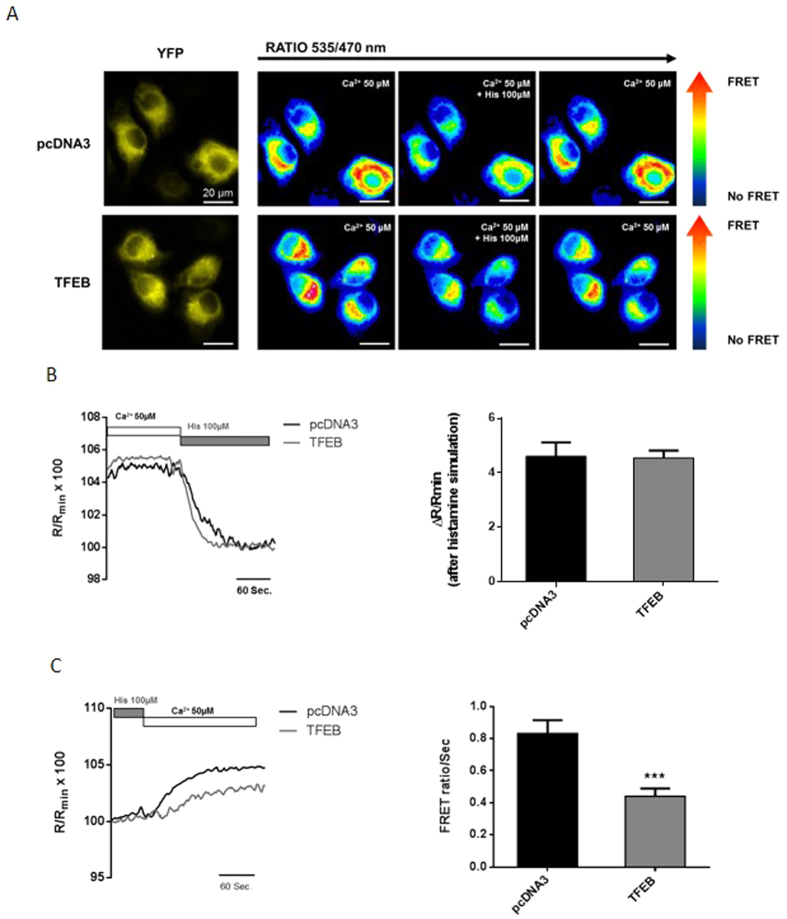
TFEB reduces Ca^2+^ re-uptake in the endoplasmic reticulum. (**A**) Representative images of D1ER probe yellow fluorescence (YFP) and FRET ratio images of HeLa cells transfected with pcDNA3 (up) or TFEB (below), before and after stimulation with histamine (100 μM) and after washout of the perfused ER Ca^2+^-releasing stimulus. (**B**) Measurements of the Δ FRET ratio (ΔR/R_min_) in HeLa cells co-transfected with pcDNA3 (control) or TFEB3xflag (TFEB) for 48 h during lysosomal emptying after histamine (100 μM) stimulation (n = 10 for each condition). (**C**) Ca^2+^ re-uptake rate measurements in pcDNA3 (control)- or TFEB3xflag (TFEB)-transfected cells during washout of the agonist (histamine 100 μM) (pcDNA3 n = 28, TFEB n = 20). Data are presented as the means ± SEM; ***p < 0.001.

Therefore, we determined whether lysosomes affect ER Ca^2+^ dynamics in response to stimuli. Reticular Ca^2+^ measurements were performed using the genetically encoded fluorescence resonance energy transfer (FRET)-based probe D1ER chameleon, which is targeted to the ER via a KDEL retention sequence^45^. This probe contains a Ca^2+^-binding domain that, once Ca^2+^ is bound, allows the highly efficient excitation transfer from a donor cyan fluorescent protein to an acceptor yellow fluorescent protein. The degree of FRET provides a ratiometric indicator of the Ca^2+^ level within the ER^46^ (Fig. 3A). We performed FRET experiments in HeLa cells transfected with D1ER. When the cells were perfused with KRB and Ca^2+^ (50 μM) at the start of the experiment, we measured the baseline FRET ratio, which corresponds to the resting Ca^2+^ level (see Fig. 3A,B and Supplementary Fig. S4A). Subsequently, we added an agonist (histamine 100 μM) that acts on Gq-coupled PM receptors triggering the production of IP3, thereby stimulating Ca^2+^ release from the ER through IP3Rs. Ca^2+^ depletion in the ER leads to the activation of Ca^2+^ channels, resulting in Ca^2+^ influx from the extracellular medium through the PM. At this point, the rate of Ca^2+^ re-uptake in the ER was measured (Fig. 3A,C and Supplementary Fig. S4A).

HeLa cells transiently overexpressing TFEB did not display any difference in ER Ca^2+^ concentration at baseline compared with control (pcDNA3) cells (Fig. 3B) (Δ FRET ratio in control HeLa cells = 4.61 ± 0.51 a.u. vs. TFEB-overexpressing HeLa cells = 4.55 ± 0.27 a.u.). These data were also confirmed through an ER-targeted aequorin-based approach. After the induction of ER Ca^2+^ emptying with ionomycin (3 μM) and EGTA (600 μM) (see Methods), the cells were perfused with Ca^2+^ at an extracellular concentration of 50 μM, and the maximum basal level of accumulated Ca^2+^ was measured (Supplementary Fig. S4B). We did not observe any difference in ER baseline Ca^2+^ levels in TFEB-overexpressing cells compared with control cells (ER Ca^2+^ concentration in control HeLa cells = 223.96 ± 3.40 μM vs. TFEB-overexpressing HeLa cells 230.75 ± 4.31 μM). However, TFEB-overexpressing cells showed a significant reduction in the Ca^2+^ re-uptake rate (Fig. 3A and C) after agonist washout compared with control cells (Ca^2+^ re-uptake rate in HeLa control cells = 0.83 ± 0.08 R/R_min_ × 100 vs. TFEB-overexpressing cells = 0.44 ± 0.05 R/R_min_ × 100), supporting the notion that TFEB-mediated SOCE inhibition could reduce Ca^2+^ refilling within the ER.

### Impairment of lysosomal activity or TFEB knockdown promotes the recovery or an increase of the Ca^2+^ re-uptake rate in the ER, respectively

We thus hypothesized that the increased quantity of lysosomes near the PM induced by TFEB overexpression might be responsible for the reduction in SOCE, thus delaying its sequestration by the ER. To confirm our hypothesis, we performed FRET measurements of Ca^2+^ reuptake to evaluate ER Ca^2+^ dynamics after pre-treatment of HeLa cells with functional inhibitors of lysosomes.

**Figure 4:**
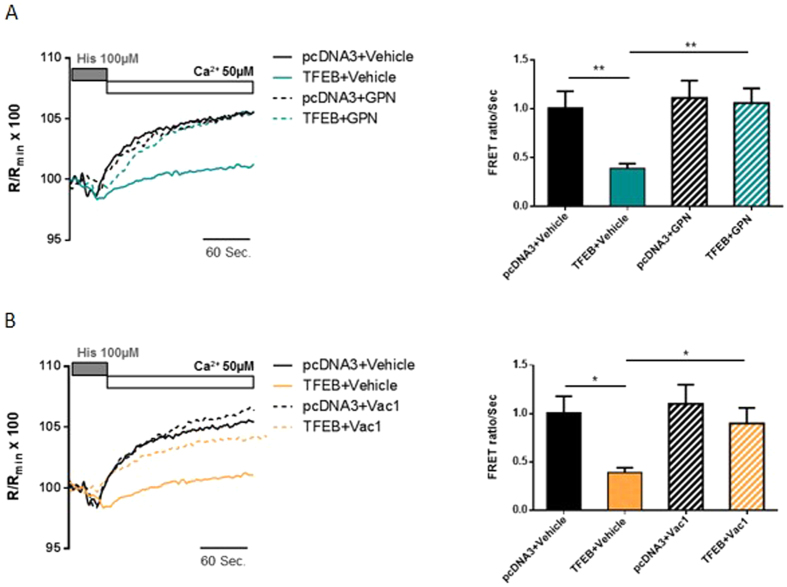
Lysosomal impairment promotes recovery of the rate of Ca^2+^ re-uptake in the endoplasmic reticulum, without affecting SOCE protein expression. (**A** and **B**) Measurement of the Ca^2+^ re-uptake rate in the ER after depletion with an agonist (histamine 100 μM) using the ER-targeted chameleon (D1ER) probe in HeLa cells co-transfected with pcDNA3 (control) or TFEB3xflag (TFEB) for 48 h. As previously described for the aequorin experiments, cells were pretreated with 200 μM GPN (**A**) for 30 min or 10 μM Vac-1 (**B**) for 1 h, or with DMSO as a control. In both experiments, the rate of Ca^2+^ re-uptake into the ER was significantly recovered in HeLa cells overexpressing TFEB (for GPN experiments: pcDNA3 + Vehicle n = 10, TFEB + Vehicle n = 11, pcDNA3 + GPN n = 9, TFEB + GPN n = 9; for Vac-1 experiments: pcDNA3 + Vehicle n = 10, TFEB + Vehicle n = 11, pcDNA3 + Vac-1 n = 9, TFEB + Vac-1 n = 13). Data are presented as the means ± SEM; *p < 0.05, **p < 0.01.

GPN treatment (200 μM for 30 min) minimized the effects of TFEB overexpression on the ER Ca^2+^ re-uptake rate (Ca^2+^ reuptake rate in untreated TFEB-overexpressing HeLa cells = 0.39 ± 0.05 R/R_min_ × 100 vs. TFEB-expressing cells + GPN = 1.06 ± 0.15 R/R_min_ × 100) (Fig. 4A). Moreover, upon Vac-1 treatment (10 μM for 1 h) (Fig. 4B), we observed a partial restoration of the ER Ca^2+^ re-uptake rate in HeLa cells expressing TFEB (Ca^2+^ re-uptake rate in untreated control cells = 1.01 ± 0.17 R/R_min_ × 100; TFEB-overexpressing untreated cells = 0.39 ± 0.05 R/R_min_ × 100; HeLa control cells treated with Vac-1 = 1.10 ± 0.20 R/R_min_ × 100; TFEB-overexpressing cells treated with Vac-1 0.90 ± 0.16 R/R_min_ × 100).

Taken together, these results confirm the effect of TFEB overexpression on the rate of ER Ca^2+^ re-uptake due to the Ca^2+^ buffering capacity of PM-located lysosomes.

To verify the effect of TFEB on SOCE modulation, we performed analogous FRET experiments in TFEB-depleted cells and measured the rate of ER Ca^2+^ re-uptake (Supplementary Fig. S5). Down-regulation of TFEB induced a significant increase in the rate of Ca^2+^ re-uptake compared to control cells (Ca^2+^ re-uptake rate in HeLa control cells = 0.07 ± 0.01 R/R_min_ × 100 vs. TFEB-silenced cells = 0.12 ± 0.02 R/R_min_ × 100), supporting a specific role for TFEB in regulating SOCE.

To further confirm our results regarding lysosome-mediated Ca^2+^ regulation and to exclude a direct effect of TFEB overexpression on SOCE molecular machinery, we first examined the protein expression levels of two main modulators of the capacitative Ca^2+^ influx pathway: STIM1 and ORAI1. Supplementary Fig. S6A showed that TFEB did not affect the expression level of either STIM1 or ORAI1. We then investigated the formation of STIM1 and ORAI1 clusters to clarify whether overexpression of TFEB can influence the contact sites between two proteins involved in the activation of SOCE. Therefore, we induced Ca^2+^ emptying from the ER under the same experimental conditions used for cytosolic Ca^2+^ measurements and assessed the number of ORAI1 clusters using the recombinant reporter ORAI1-EYFP. After treatment with thapsigargin; (200 nM) in Ca^2+^-free medium (KRB/EGTA), the number of clusters of ORAI1 increased considerably (see Supplementary Fig. S6B) in both control and TFEB-overexpressing HeLa cells (n° of ORAI1 PM clusters × cell after SOCE induction in pcDNA3 HeLa cells = 82.56 ± 6.44 vs. TFEB-overexpressing HeLa cells = 80.77 ± 4.50), but no significant differences were detected between pcDNA3 and TFEB-transfected cells.

Moreover, we examined the formation of STIM1 puncta resulting from the same treatment (see above) using the recombinant reporter STIM1-ECFP; again, we did not observe significant differences between TFEB-expressing and control HeLa cells (see Supplementary Fig. S6C) (no. of STIM1 PM cluster × cell after SOCE induction in pcDNA3-transfected cells = 94.54 ± 11.40 vs. TFEB-overexpressing cells = 96.07 ± 7.27), supporting a crucial role for the lysosomal compartment in the TFEB-dependent regulation of Ca^2+^ influx.

### Modulation of TFEB expression affects lysosomal Ca^2+^ contents after SOCE induction

As noted above, the increase in the biogenesis of lysosomes and their localization to the PM, induced by overexpression of TFEB affected SOCE, leading to lower calcium uptake in the cytosol and a reduced rate of Ca^2+^ charging of the ER. Conversely, downregulation of TFEB reduced the number of lysosomes and their distribution in the proximity of plasma membrane, associated with increases in cytosolic Ca^2+^ uptake and the Ca^2+^ re-uptake rate of the ER during capacitative Ca^2+^ influx. We thus speculated that lysosomes proximal to the PM take up Ca^2+^ imported into the cell by SOCE, depriving the ER of this ion.

**Figure 5:**
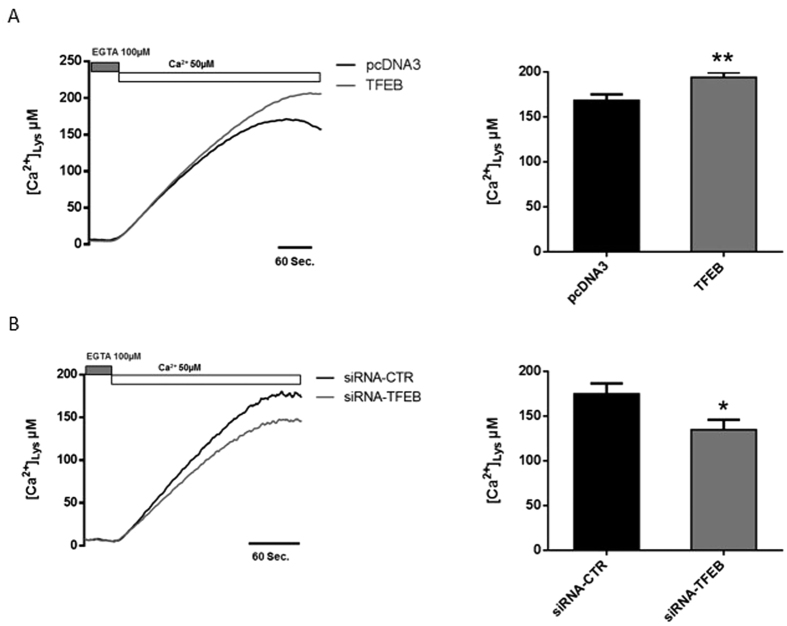
Lysosomal Ca^2+^ content is affected by TFEB expression. (**A**) Ca^2+^ reuptake measured with lysosomal aequorin in control HeLa cells (pcDNA3) or HeLa cells overexpressing TFEB for 48 h, after Ca^2+^ (50 μM) administration (pcDNA3 n = 9, TFEB n = 11). (**B**) Capacitative Ca^2+^ entry was measured using lys-Aeq in HeLa cells with the control siRNA (siRNA-CTR) or siRNA targeting TFEB (siRNA-TFEB) after Ca^2+^ (50 μM) administration (siRNA-CTR n = 8, siRNA-TFEB n = 10). Data are presented as the means ± SEM; *p < 0.05, **p < 0.01.

To confirm our hypothesis, we directly assessed lysosomal Ca^2+^ concentrations ([Ca^2+^]_Lys_) using an aequorin fused with the full-length cathepsin D protein, which specifically targets the probe to lysosomes^21^. If the hypothesis is correct, we expected that TFEB overexpression, a condition able to reduce SOCE would increase [Ca^2+^]_Lys_. To induce capacitative Ca^2+^ influx via SOCE activation, HeLa cells were treated with ionomycin (3 μM) for 1 h in a Ca^2+^-free medium (KRB/EGTA) during aequorin reconstitution with coelenterazine to encourage depletion of ER and lysosomal Ca^2+^ stores. When the release of stored Ca^2+^ was complete, Ca^2+^ (CaCl_2_ 50 μM) was added to the KRB perfusion. In our experimental setting, HeLa cells overexpressing TFEB (Fig. 5A) displayed a significant increase in lysosomal Ca^2+^ compared with control cells ([Ca^2+^]_Lys_ in control HeLa cells = 168.80 ± 6.59 μM vs. TFEB-overexpressing HeLa cells 194.17 ± 5.80 μM). Accordingly, we detected a significant reduction in [Ca^2+^]_Lys_ in TFEB-silenced cells compared to siRNA control cells ([Ca^2+^]_Lys_ in siRNA control HeLa cells = 175.26 ± 11.58 μM vs. siRNA-TFEB HeLa cells 134.91 ± 11.26 μM) (Fig. 5B), corroborating the role of TFEB in the regulation of SOCE by specifically modulating the lysosomal Ca^2+^-buffering capacity.

As previously observed, Vac-1 treatment affected the capacity of lysosomes to interact with other organelles^43^, thus reducing the fusion of lysosomes with the PM^44^. To clarify whether the lack of proximity of lysosomes to the PM under Vac-1 treatment might influence SOCE, we directly assessed the lysosomal Ca^2+^ concentration in HeLa cells transiently transfected with empty vector (pcDNA3) or TFEB after pretreatment with Vac-1 (10 μM for 1 h) or DMSO (vehicle). Under experimental conditions including only the vehicle, we observed maintenance of the differences in lysosomal Ca^2+^ between control and TFEB-overexpressing samples (see Supplementary Fig. S7). Importantly, Vac-1 treatment significantly reduces lysosomal Ca^2+^-buffering capacity exclusively in HeLa cells expressing TFEB (lysosomal Ca^2+^ concentration in TFEB-overexpressing HeLa treated with vehicle = 183.82 ± 10.46 μM vs. TFEB-overexpressing Vac-1-treated cells = 137.78 ± 13.17 μM), without altering the lysosomal Ca^2+^ levels of control cells (lysosomal Ca^2+^ concentration in pcDNA3 HeLa cells treated with DMSO = 146.24 ± 6.40 μM vs. pcDNA3 Vac-1-treated cells = 127.51 ± 7.02 μM). Overall, these data suggest that the effect of TFEB activity on SOCE and on lysosomal Ca^2+^ refilling is strictly dependent on both lysosomal activity and the PM-specific positioning of lysosomes.

As previously demonstrated by Medina *et al*.^20^, lysosomal Ca^2+^ release via MCOLN1 controls the activity of the phosphatase calcineurin during starvation. This pathway activates TFEB, thus promoting its nuclear translocation and lysosomal compartment expansion^30^.

To validate our experimental setup, we first performed immunofluorescence staining of HeLa cells overexpressing TFEB before and after 6 h of starvation. Calcineurin activity was inhibited with cyclosporin A (CsA) to prevent TFEB translocation to the nucleus. As expected, analysis of the TFEB distribution revealed a nuclear-enriched pattern upon starvation (Supplementary Fig. S8A) (TFEB nuclear localization of fed HeLa cells treated with DMSO = 0.96 ± 0.04 vs. starved HeLa cells treated with DMSO = 2.06 ± 0.11) that was abolished by CsA treatment (TFEB nuclear localization of starved HeLa cells treated with DMSO = 2.06 ± 0.11 vs. starved HeLa cells treated with CsA = 1.07 ± 0.02). Then, to investigate the possible role of the lysosomes/calcineurin/TFEB axis in SOCE modulation, we assessed the level of cytosolic Ca^2+^ entering via SOCE in pcDNA3 and TFEB-overexpressing HeLa cells under the same condition described above. Control cells showed no significant differences in SOCE either during starvation (Supplementary Fig. S8B) (cytosolic Ca^2+^ uptake fed pcDNA3 HeLa cells treated with DMSO = 21.32 ± 1.65 a.u.c.; starved pcDNA3 HeLa cells treated with DMSO = 20.82 ± 2.94 a.u.c.) or after CsA treatment (fed pcDNA3 HeLa cells treated with CsA = 18.25 ± 2.04; starved pcDNA3 HeLa cells treated with CsA = 21.01 ± 1.28 a.u.c.). Intriguingly, TFEB-mediated reduction of SOCE (cytosolic Ca^2+^ uptake by fed pcDNA3 HeLa cells treated with DMSO = 21.32 ± 1.65 a.u.c. vs. fed TFEB HeLa cells treated with DMSO = 13.95 ± 1.60 a.u.c.) was amplified by starvation (fed TFEB HeLa cells treated with DMSO = 13.95 ± 1.60 a.u.c. vs. starved TFEB HeLa cells treated with DMSO = 3.74 ± 1.35 a.u.c.), and this effect was completely prevented by CsA treatment during starvation (starved TFEB HeLa cells treated with DMSO = 3.74 ± 1.35 a.u.c. vs starved TFEB-overexpressing HeLa cells treated with CsA = 14.35 ± 0.78 a.u.c.). These data indicate that increasing the nuclear localization of TFEB through starvation further stimulates lysosomal biogenesis^30^, thereby enhancing the role of lysosomes in the modulation of SOCE.

## Discussion

For a long time, lysosomes have been considered to be merely cellular ‘incinerators’ involved in the degradation and recycling of cellular waste^47^. However, there is now compelling evidence that lysosomes have a much broader function and may assume a pivotal role in intracellular signaling. Indeed, in a recent study, Kilpatrick and coworkers speculate that TRPML1 activation evokes a global Ca^2+^ signal that originates from the ER, lysosomes, and influx from the extracellular environment, suggesting a possible location of a small portion of TRPML1 to the PM^48^. Lysosomes can accumulate Ca^2+^ released from the ER^22^,^43^, and the observation that these two organelles are intimately associated sheds new light on the multiple functions of lysosomes and on their important roles in cellular homeostasis.

In addition, the identification of TFEB as a master regulator of lysosomal biogenesis and autophagy has revealed how the lysosome adapts to environmental cues such as nutrient availability. Targeting TFEB may provide a novel therapeutic strategy for modulating lysosomal functions in human diseases.

We investigated the role of TFEB in orchestrating lysosomal biogenesis and the role of lysosomes in Ca^2+^ homeostasis. Through modulation of TFEB expression in HeLa cells, we attempted to better understand the role of lysosomes in maintaining Ca^2+^ homeostasis. We showed that increases in the number of lysosomes and their proximity to the PM upon TFEB overexpression strongly influence Ca^2+^ entry across the PM (Fig. 1) and lysosomal calcium replenishment (Fig. 5A) after SOCE activation. Using pharmacological approaches to alter both the activity and distribution of lysosomes (Fig. 2B and D), we showed that the TFEB-dependent reduction of SOCE is dependent on lysosomal dynamics. In particular, the reduction in interacting membrane surface after Vac-1 treatment suggests that this buffering role is explained by the proximity of the PM. Conversely, transient TFEB knockdown reduces the number of lysosomes and their localization to the PM, increasing cytosolic Ca^2+^ accumulation (see Supplementary Fig. S3D), the reticular Ca^2+^ replenishment rate (see Supplementary Fig. S5) and lysosomal buffering capacity in a location-dependent manner (Fig. 5B).

In recent studies, Lopez-Sanjurjo and co-workers^43^,^49^ revealed that i) the close association between lysosomes and the ER allows lysosomes to selectively accumulate Ca^2+^ released from the ER and that ii) disruption of lysosomal Ca^2+^ uptake does not affect SOCE. The results of the present study confirm these findings; alteration of the lysosomal compartment by different pharmacological approaches did not compromise SOCE in control cells (Fig. 2B and D). However, we observed a restoration of capacitative influx only in TFEB-overexpressing cells treated with both GPN and Vac-1 (Figs 2B,D and 4). Therefore, under physiological conditions, lysosomes are prone to sequestering Ca^2+^ released by the ER due to the juxtaposition between the two organelles, whereas under specific situations such as TFEB overexpression that undermine the redistribution of lysosomes, different Ca^2+^ sources might represent the primary target of lysosomal Ca^2+^ buffering activity. Interestingly, lysosomes located at different intracellular regions exhibit high heterogeneity^50^. Peripheral lysosomes are more alkaline (pH values ≥6) than juxtanuclear lysosomes due to increased passive (leak) permeability of H^+^ and reduced vacuolar V-ATPase activity. Nevertheless, lysosomal buffering capacity appears to be comparable between central and marginal lysosomes, whereas peripheral lysosomes display reduced cathepsin L activity. Based on the widely accepted view that lysosomal acidification drives Ca^2+^ into lysosomes^43^, it is reasonable to assume that peripheral lysosomes possess a decreased capacity for Ca^2+^ uptake due to their higher pH. However, a recent paper by Garrity *et al*. contests the ‘pH hypothesis’ of lysosomal Ca^2+^ accumulation by elegantly demonstrating that under normal conditions, lysosome Ca^2+^ stores are refilled from the ER through IP3 receptors in a manner independent of lysosomal pH^22^. Moreover, in secretory granules and the ER, increasing the luminal pH raised the Ca^2+^ buffering capacity of both compartments^51^. Taken together, these findings suggest that the primary effects of TFEB expression on extracellular Ca^2+^ entry might be specifically due to the lower acidification of the lysosomes closest to the PM, which in turn might result in a higher Ca^2+^ buffering capacity.

The principal activity of perimetral lysosomes is fusion with the PM to complete the process of exocytosis in a Ca^2+^-dependent manner^52^. Lysosomes likely provide the Ca^2+^ required for lysosomal fusion^52^. Interestingly, TFEB modulates exocytosis, causing increased expression/activity of tethering factors or proteins involved in lysosome mobility, docking^27^ and fusion to the PM^30^. In particular, upregulation of the lysosomal Ca^2+^ channel TRPML1 triggers the final fusion of these organelles through localized Ca^2+^ release^30^. Therefore, the buffering capacity of lysosomes close to the PM, which we observed in TFEB-overexpressing cells, could be critical for promoting lysosomal Ca^2+^ refilling, thereby favoring lysosomal exocytosis.

Moreover, a recent study by Medina *et al*.^20^ revealed that lysosomes can release Ca^2+^ via MCOLN1 during starvation. This lysosomal Ca^2+^ efflux triggers the phosphatase calcineurin, which de-phosphorylates TFEB, thus promoting its nuclear translocation and autophagy. In this work, we demonstrate that TFEB increases [Ca^2+^]_Lys_, which would, in turn, potentiate calcineurin activation during starvation. It is therefore proposed that TFEB modulation of SOCE and [Ca^2+^]_Lys_ stimulates a positive feedback loop that maximizes the TFEB-mediated control of lysosomal biogenesis and recruitment to the plasma membrane. In support of this model, we showed that starvation exacerbates the role of lysosomes in the modulation of SOCE, and this phenomenon is completely prevented by CsA-mediated inhibition of calcineurin (see Supplementary Fig. S8).

Importantly, the TFEB-mediated regulation of SOCE was observed only in the presence of a low extracellular Ca^2+^ concentration (50 μM) (see Supplementary Fig. S2). Although studies on the role of lysosomes in Ca^2+^ signaling have greatly increased in number over the last decade, the nature of the exchanger/channel responsible for lysosomal Ca^2+^ uptake remains unknown. Based on our data, we suggest that the lysosomal compartment represents a system that responds well to low concentrations of Ca^2+^ and has a high affinity for the cation, as speculated in other works^12^,^43^.

In conclusion, we provide new evidence for the role of TFEB in shaping extracellular Ca^2+^ entry through the re-distribution of the lysosomal compartment to the PM. These findings not only describe TFEB as a new regulator of intracellular Ca^2+^ homeostasis but also propose lysosomal control of SOCE as an important aspect of lysosomal function and cellular health.

## Methods

### Reagents and solutions

The following chemicals were used: di-peptide glycyl-L-phenylalanine 2-naphtylamide (GPN; Bachem), vacuolin 1 (Vac-1; Calbiochem), thapsigargin (Sigma-Aldrich), ethylene glycol-bis (β-aminoethyl ether)-N,N,N′,N′-tetraacetic acid (EGTA; Sigma-Aldrich), cyclosporin a (CsA; Sigma-Aldrich).

### Cell culture and transient transfection

HeLa cells were grown in Dulbecco’s modified Eagle’s medium (DMEM) supplemented with 10% fetal bovine serum (FBS; Life Technologies), 2 mM L-glutamine, and 100 U/ml penicillin (EuroClone), and 100 mg/ml streptomycin (EuroClone) in 75 cm^2^ Corning flasks. All cells were maintained at 37 °C under 90% relative humidity with 5% CO_2_. Before transfection, cells were seeded onto 13 mm glass coverslips for intracellular Ca^2+^ measurements with aequorin and onto 24 mm glass coverslips for microscopic analysis. Cells were grown to 70% confluence and then transfected for 12 h at 37 °C for transient plasmid overexpression. Transfection was performed via the standard Ca^2+^phosphate procedure^53^. All experiments were performed 48 h after transfection.

For siRNA experiments, cells were seed onto 13 mm glass coverslips for intracellular Ca^2+^ measurements with aequorin and onto 24 mm glass coverslips for microscopic analysis. siRNAs were transfected with Lipofectamine RNAiMAX (Invitrogen) using a reverse transfection protocol. After 24 h, the cells were transfected for 12 h at 37 °C to achieve transient plasmid overexpression, via the standard Ca^2+^ phosphate procedure^53^, and siRNA experiments were performed 48 h after the last transfection.

### Aequorin measurements

The probes used in these experiments were chimeric aequorins targeted to the cytosol (cyt-Aeq), PM (PM-Aeq), ER (ER-Aeq) and lysosomes (lys-Aeq). To evaluate the capacitative Ca^2+^ entry current in the experiments with cyt-Aeq, at 48 h post-transfection, the coverslips were incubated with 5 μM coelenterazine (Fluka) for 1.5 h in Krebs–Ringer modified buffer (KRB; 125 mM NaCl, 5 mM KCl, 1 mM Na_3_PO_4_, 1 mM MgSO_4_, 5.5 mM glucose, and 20 mM 4-(2-hydroxyethyl)-1-piperazineethanesulfonic acid [HEPES], pH 7.4, at 37 °C) supplemented with 1 mM CaCl_2_. For the PM-Aeq experiments, the coverslips were incubated in KRB supplemented with 5 μM coelenterazine and 100 μM EGTA for 1 h.

Then, to induce ER Ca^2+^ emptying, cells were incubated with 200 nM thapsigargin (SERCA inhibitor) and 1 mM EGTA in Ca^2+^-free KRB for 15 min at 4 °C. A coverslip with transfected cells was subsequently placed in a perfused thermostat-controlled chamber located in close proximity to a low-noise photomultiplier with a built-in amplifier/discriminator. After 30″ of perfusion with KRB supplemented with 100 μM EGTA, Ca^2+^ was added to KRB, as specified in the figure legends. All experiments were terminated by lysing cells with X-100 Triton in a hypotonic Ca^2+^-containing solution (10 mM CaCl_2_ in H_2_O), thus discharging the remaining aequorin pool.

To reconstitute ER-Aeq and lys-Aeq with a high efficiency, the luminal [Ca^2+^] of the intracellular Ca^2+^ stores was first reduced by incubating the cells for 45 min at 4 °C in KRB supplemented with 5 μM coelenterazine N, the Ca^2+^ ionophore ionomycin, and 600 μM EGTA. After incubation, the cells were extensively washed with KRB supplemented with 2% bovine serum albumin and 2 mM EGTA before luminescence measurements were initiated. After 180″ of perfusion with KRB supplemented with 100 μM EGTA, 50 μM Ca^2+^ was added to KRB, as specified in the figure legends. For the ER-Aeq experiments, the agonist (histamine at 100 μM) was added to the same medium after a Ca^2+^ plateau was reached.

All experiments were terminated by lysing cells with X-100 Triton in a hypotonic Ca^2+^-containing solution (10 mM CaCl_2_ in H_2_O), thus discharging the remaining aequorin pool.

For the experiments with GPN, the cells were pretreated for 30′ during the period of incubation using coelenterazine and 200 μM GPN to induce emptying of the ER or DMSO in control cells. For experiments with Vac-1, the cells were pretreated with coelenterazine for 1 h during the period of incubation, and emptying of the ER was induced with 10 μM Vac1; control cells were treated with DMSO.

The output of the discriminator was captured with a Thorn EMI photon-counting board and stored in an IBM-compatible computer for further analyses. The aequorin luminescence data were calibrated into [Ca^2+^] values offline, using a computer algorithm based on the Ca^2+^ response curve of wild-type aequorin. Further experimental details were previously described^35^.

### Acquisition of fluorescence microscopy observations of the treatments

HeLa cells expressing lysosomal-targeted GFP (LAMP1-GFP) were stained with LysoTracker RED (Invitrogen) and treated with GPN, Vac1 or vehicle alone, as indicated in the text and figures. For all treated cells, digital images were acquired in a 37 °C thermostat-controlled incubation chamber with a confocal microscope (Zeiss LSM510), using a 40 × 1.4 NA Plan-Apochromat oil-immersion objective. The acquired images were subsequently analyzed using the open source software Fiji.

### Measurements of ER Ca^2+^ dynamics with D1ER

Luminal Ca^2+^ dynamics were measured using single-cell Ca^2+^ imaging and the Ca^2+^-sensitive FRET (fluorescence resonance energy transfer)-based chameleon protein D1ER^45^. HeLa cells were co-transfected with D1ER and TFEB-3xFLAG or empty vector (pcDNA3). After 40 h, coverslips were placed in 1 ml of KRB, and images were captured with METAFLUOR 7.0 Software (Universal Imaging) at λ_excitation_ = 430 nm and λ_emission_ = 470 and 535 nm every 2 s using a Zeiss Axiovert 200 M inverted microscope equipped with a C-Apochromat 40x/1.2 W CORR objective and a cooled CCD camera (Photometrics). CFP emission and yellow fluorescent protein (YFP) FRET emission were alternately collected at 470 and 535 nm, respectively. The FRET signal was normalized to the CFP emission intensity, and changes in ER Ca^2+^ were expressed as the ratio of the emissions at 535 and 470 nm. Cells were perfused with KRB with 50 μM Ca^2+^ at the beginning of the experiment, and the baseline of the FRET ratio was measured, which corresponds to resting Ca^2+^. Then, 100 μM histamine was added to the perfusion, thus stimulating Ca^2+^ release from the ER through IP3Rs (R_min_). Finally, the agonist as washed out using KRB with 50 μM Ca^2+^, and the rate of Ca^2+^ re-uptake in the ER was measured. Cells were pre-treated with GPN, Vac1 or DMSO (vehicle), as previously described for cytosolic Ca^2+^ measurement with aequorin and indicated in the text and figures.

### Statistical analysis

Statistical analyses were performed using an unpaired two-tailed t-test (two groups) or one-way ANOVA with Tukey correction (for groups of three or more). For grouped analyses, two-way ANOVA was performed; p < 0.05 was considered significant. All data are reported as the means ± SEs.

## Additional Information

**How to cite this article**: Sbano, L. *et al*. TFEB-mediated increase in peripheral lysosomes regulates store-operated calcium entry. *Sci. Rep.* **7**, 40797; doi: 10.1038/srep40797 (2017).

**Publisher's note:** Springer Nature remains neutral with regard to jurisdictional claims in published maps and institutional affiliations.

## Supplementary Material

Supplementary Information
