# Supplementary material for: TFEB-mediated increase in peripheral lysosomes regulates store-operated calcium entry
Source: Sci Rep. 2017 Jan 13;7:40797. doi: 10.1038/srep40797 (PMC5233950; doi:10.1038/srep40797)

TFEB-mediated increase in peripheral lysosomes regulates Store Operated Calcium Entry

Luigi Sbano, Massimo Bonora, Saverio Marchi, Federica Baldassari, Diego L. Medina, Andrea Ballabio, Carlotta Giorgi and Paolo Pinton

Supplementary information

Methods

Immunoblotting

For immunoblotting, cells were scraped into ice-cold, phosphate buffered saline (PBS) and lysed in a buffer containing 50 mM Tris HCl pH 7.4, 150 mM NaCl, 1% Triton X-100, 0.2% SDS, protease and phosphatase inhibitor cocktail. After 30 min of incubation on ice and centrifugation at 2,500 rpm at 4°C for 5 min, proteins were quantified by the Lowry method, and 10 µg of each sample was loaded onto a Novex NuPage Bis-Tris 4–12% precast gel (Life Technologies). The separated proteins were transferred to nitrocellulose membranes. After incubation with TBS–Tween-20 (0.05%) supplemented with 5% non-fat powdered milk for 1 h to saturate unspecific binding sites, the membranes were incubated overnight with primary antibodies. Detection was achieved using appropriate horseradish peroxidase-labeled secondary antibodies (Santa Cruz Biotechnology), followed by biochemiluminescence (ThermoScientific) using an ImageQuant LAS4000 (GE Healthcare).

Antibodies

The following primary antibodies were used for Western blotting: rabbit anti-FLAG [F7425] (1:2,000) and mouse anti-b-actin [A1978] (1:10,000) from Sigma-Aldrich; rabbit anti-GAPDH [#2118] (1:5,000) and rabbit anti-TFEB [#4240] (1:1000) from Cell Signaling; rabbit anti-STIM1 [sc-68897] (1:1,000) and rabbit anti-ORAI1 [sc-68895] (1:1,000) from Santa Cruz Biotechnology.

Analysis of lysosomal morphology

HeLa cells expressing a lysosomal-targeted variant of GFP (LAMP1-GFP) were imaged using an IX-81 automated epifluorescence microscope (Olympus) equipped with a 60× oil-immersion objective (N.A. 1.35, from Olympus) and an ORCA-R2 CCD camera (Hamamatsu Photonics K.K.).

Selected cells were followed over time, and z-stacks were subjected to digital deconvolution using a Wiener deconvolution filter and a theoretical point-spread function provided by Xcellence software (Olympus). GFP-positive objects were quantified using BITPLANE Imaris 4.

Cell treatment in the starvation experiment

For starvation experiments, cells were cultured in the following media for 6 h: (normal) DMEM high glucose supplemented with 10% FBS; (starvation) HBSS with Ca^{2+} and Mg^{2+} supplemented with 10 mM HEPES. During starvation, cells were treated with CsA (10 μM) or DMSO (vehicle) to inhibit the phosphatase calcineurin.

Immunofluorescence

Cells, transfected or treated as described, were washed with PBS and fixed with 4% formaldehyde for 10 min at room temperature. After washing three times with PBS, the cells were permeabilized with 0.1% Triton X-100 in PBS (PBST) at room temperature for 10 min and blocked with PBST containing 5% BSA at room temperature for 1 h. The cells were incubated with an anti-FLAG primary antibody (Sigma-Aldrich, dilution 1:100) in PBST containing 5% BSA overnight at 4°C, washed three times with PBS, and then incubated with appropriate isotype-matched, AlexaFluor 488-conjugated secondary antibodies (Life Technologies, dilution 1:1000) at room temperature for 1 h. Digital images were acquired using a confocal microscope (Zeiss LSM510) with a 63 × 1.4 NA Plan-Apochromat oil-immersion objective and analyzed using Fiji open-source software.

Figure legends

Fig. S1. Transient overexpression of TFEB increased the lysosomal network in HeLa cells.

(A) Representative immunoblot images of TFEB protein abundance in HeLa cells transfected for 48 h with the empty vector (pCDNA3) or plasmid encoding TFEB 3xflag (TFEB). (B) Representative images of lysosomal morphology and quantification of lysosomal number in HeLa cells co-transfected for 48 h with LAMP1-GFP (green) and pcDNA3 (Control) or TFEB 3xflag (TFEB) (pcDNA3 n = 27, TFEB n = 28). Data are presented as the means ± SEM, $^{**}p<0.01$.

Fig. S2. TFEB overexpression modulated capacitative Ca^{2+} entry at low extracellular Ca^{2+} concentrations.

Cytosolic Ca^{2+} measurements of capacitative Ca^{2+} entry measured by cytosolic aequorin in HeLa cells transfected with pcDNA3 or TFEB 3xflag (TFEB). After intracellular Ca^{2+} store depletion, 1 mM Ca^{2+} was perfused in solution (n = 12 for each experiment). Data are presented as the means ± SEM. a.u.c. = area under the curve.

Fig. S3. Downregulation of TFEB decreased the lysosomal network and lysosomal localization to the PM, thereby increasing capacitative Ca^{2+} entry in HeLa cells.

(A) Representative immunoblot images of TFEB protein abundance in HeLa cells transfected for 72 h with control siRNA (siRNA-CTR) or siRNA for TFEB (siRNA-TFEB). (B) Representative images of lysosomal morphology and quantification of lysosomal number in HeLa cells transfected for 48 h with LAMP1-GFP (green) and silenced for 72 h with control siRNA (siRNA-CTR) or siRNA for TFEB (siRNA-TFEB) (n = 34 for each condition). (C) Representative images and quantification of lysosomal distance to the plasma membrane normalized on the cellular area in HeLa cells transfected for 48 h with LAMP1-GFP (green) and silenced for 72 h with siRNA control (siRNA-CTR) or siRNA for TFEB (siRNA-TFEB); the plasma membrane is stained with FM4-64fx

dye (red) (siRNA-CTR n = 17, siRNA-TFEB n = 11). (D) Cytosolic Ca^{2+} measurements of capacitative Ca^{2+} entry measured by cytosolic aequorin in HeLa cells transfected with control siRNA (siRNA-CTR) or siRNA for TFEB (siRNA-TFEB). After intracellular Ca^{2+} store depletion, 50 μM Ca^{2+} was perfused in solution (siRNA-CTR n = 23, siRNA-TFEB n = 16). Data are presented as the means ± SEM; *$p<0.05$, ***$p<0.001$. a.u.c. = area under the curve.

Fig. S4. TFEB overexpression did not alter the basal ER Ca^{2+} concentration.

(A) Representation of phases of FRET experiments in HeLa cells transfected with the ER-targeted Cameleon (D1ER) probe to directly measure $[Ca^{2+}]_{ER}$. Ca^{2+} was depleted from the ER with agonist (histamine 100 μM) and subsequently stimulated with 50 μM Ca^{2+} to induce ER Ca^{2+} re-uptake. (B) The ER Ca^{2+} concentration measured with ER-targeted aequorin in control (pcDNA3) or overexpressing TFEB 3xflag (TFEB) HeLa cells in the presence of a low extracellular Ca^{2+} concentration ($CaCl_2$ 50 μM). Administration of histamine (100 μM) resulted in comparable ER emptying under both conditions (n = 10 for each condition). Data are presented as the means ± SEM.

Fig. S5. Transient TFEB downregulation increased Ca^{2+} re-uptake by the endoplasmic reticulum.

Ca^{2+} re-uptake rate measurement using the ER-targeted Cameleon (D1ER) probe in HeLa cells transfected for 72 h with control siRNA (siRNA-CTR) or siRNA for TFEB (siRNA-TFEB) during washout of agonist (histamine 100 μM) (pcDNA3 n = 41, TFEB n = 33). Data are presented as the means ± SEM; **$p<0.01$.

Fig. S6. TFEB overexpression did not influence STIM1 and ORAI1 expression or punctae formation.

(A) Representative immunoblot images of protein expression levels of STIM1 and ORAI1 in HeLa cells transfected for 48 h with a plasmid expressing pcDNA3 (Control) or TFEB 3xflag (TFEB). (B) ORAI1-YFP representative images and ORAI1 clusters quantification of HeLa cells transfected with a plasmid expressing pcDNA3 (Control) or TFEB 3xflag (TFEB) before and after treatment with thapsigargin (200 nM) for 15 min in a Ca^{2+}-free medium (KRB/EGTA) (pcDNA3 n = 16, TFEB n = 13). (C) STIM1-YFP representative images and STIM1 clusters quantification of HeLa cells transfected with a plasmid expressing pcDNA3 (Control) or TFEB 3xflag (TFEB) before and after treatment with thapsigargin (200 nM) for 15 min in a Ca^{2+}-free medium (KRB/EGTA) (pcDNA3 n = 13, TFEB n = 14). Data are presented as the means ± SEM.

Fig. S7. Vac-1 treatment reduced lysosomal Ca^{2+} in TFEB-overexpressing cells.

(A) Lysosomal Ca^{2+} uptake measured using lysosomal aequorin in HeLa cells transfected with pcDNA3 (control) or with TFEB3xflag (TFEB) and pretreated with 10 μM Vac-1 or DMSO (vehicle) for 1 h. After intracellular Ca^{2+} store depletion, the cells were perfused with 50 μM Ca^{2+} (pcDNA3 + Vehicle n = 12, TFEB + Vehicle n = 12, pcDNA3 + Vac-1 n = 9, TFEB + Vac-1 n = 9). Data are presented as the means ± SEM; *$p<0.05$, **$p<0.01$.

Fig. S8. The calcineurin/TFEB pathway modulates SOCE in TFEB-overexpressing HeLa cells.

(A) Representative immunofluorescence images and quantification of TFEB nuclear localization (green) on the total TFEB fraction of HeLa cells overexpressing TFEB 3xflag under basal conditions (fed) of after 6 h of starvation (starved), treated with vehicle (DMSO) or CsA (10 μM). The cells were also transfected with the H2B-RFP construct to mark the nucleus (red) (Fed + Vehicle n = 29, Fed + CsA n = 28, Starved + Vehicle n = 18, Starved + CsA n = 45). (B) Cytosolic Ca^{2+} measurements of capacitative Ca^{2+} entry measured using cytosolic aequorin in HeLa cells transfected with pcDNA3 or TFEB 3xflag (TFEB) under basal conditions (fed) of after 6 h of

starvation (starved), treated with vehicle (DMSO) or CsA (10 μM). After intracellular Ca^{2+} store depletion, 50 μM Ca^{2+} was perfused in solution (for pcDNA3 samples: Fed + Vehicle n = 7, Fed + CsA n = 8, Starved + Vehicle n = 7, Starved + CsA n = 8; for TFEB samples: Fed + Vehicle n = 8, Fed + CsA n = 7, Starved + Vehicle n = 8, Starved + CsA n = 7). Data are presented as the means ± SEM; $**p<0.01$, $****p<0.0001$. a.u.c. = area under the curve.

Supplementary Figure S1.

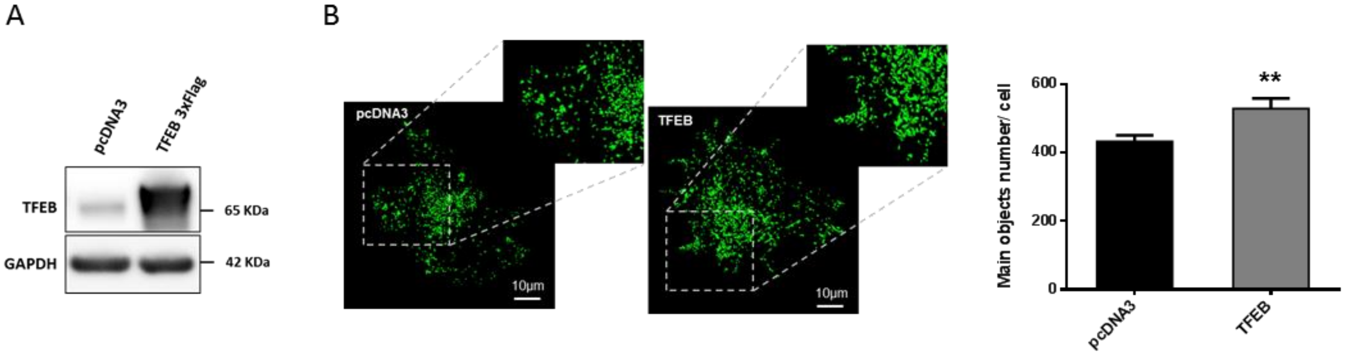

Supplementary Figure S2.

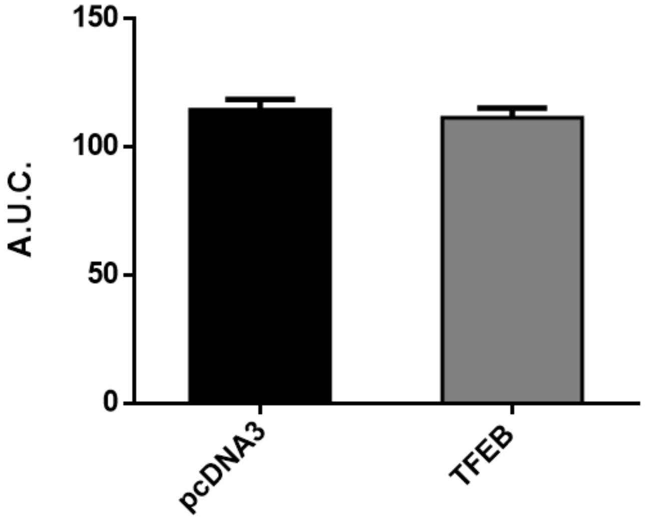

Supplementary Figure S3.

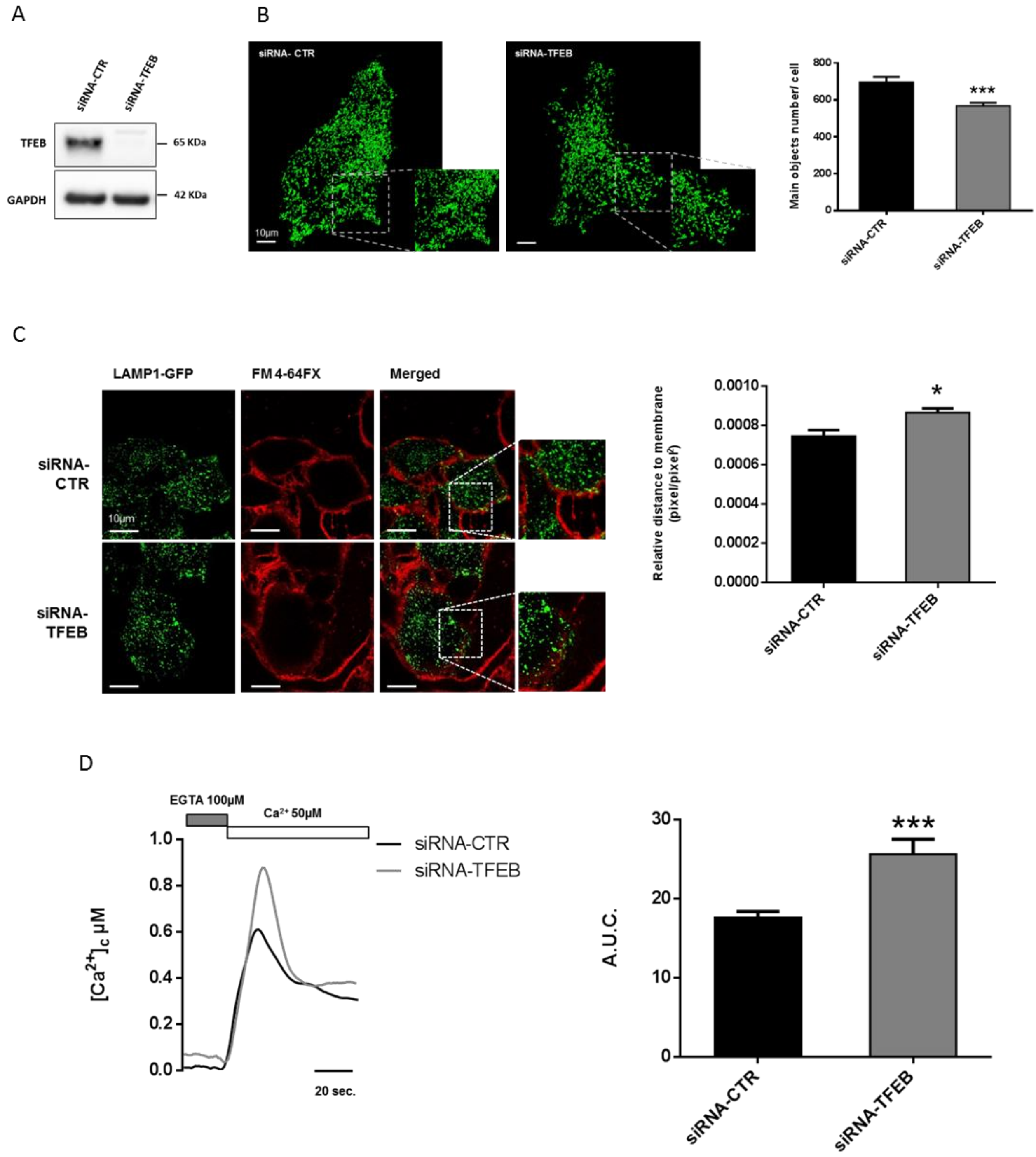

Supplementary Figure S4.

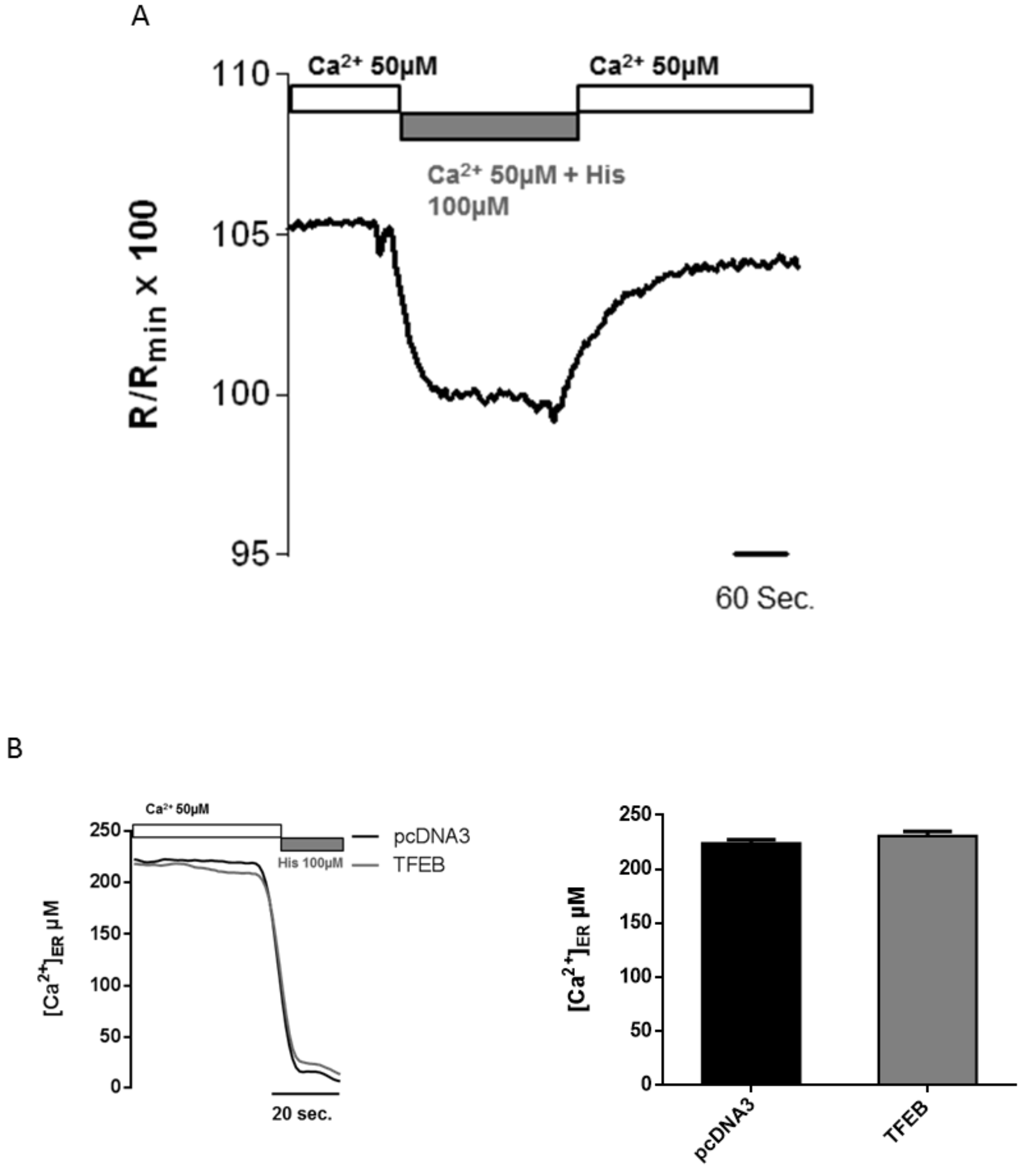

Supplementary Figure S5.

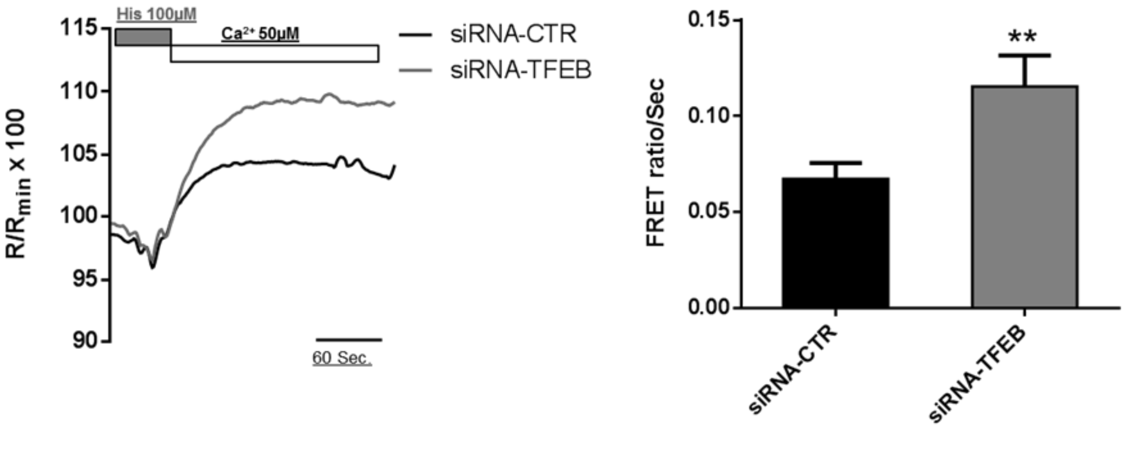

Supplementary Figure S6.

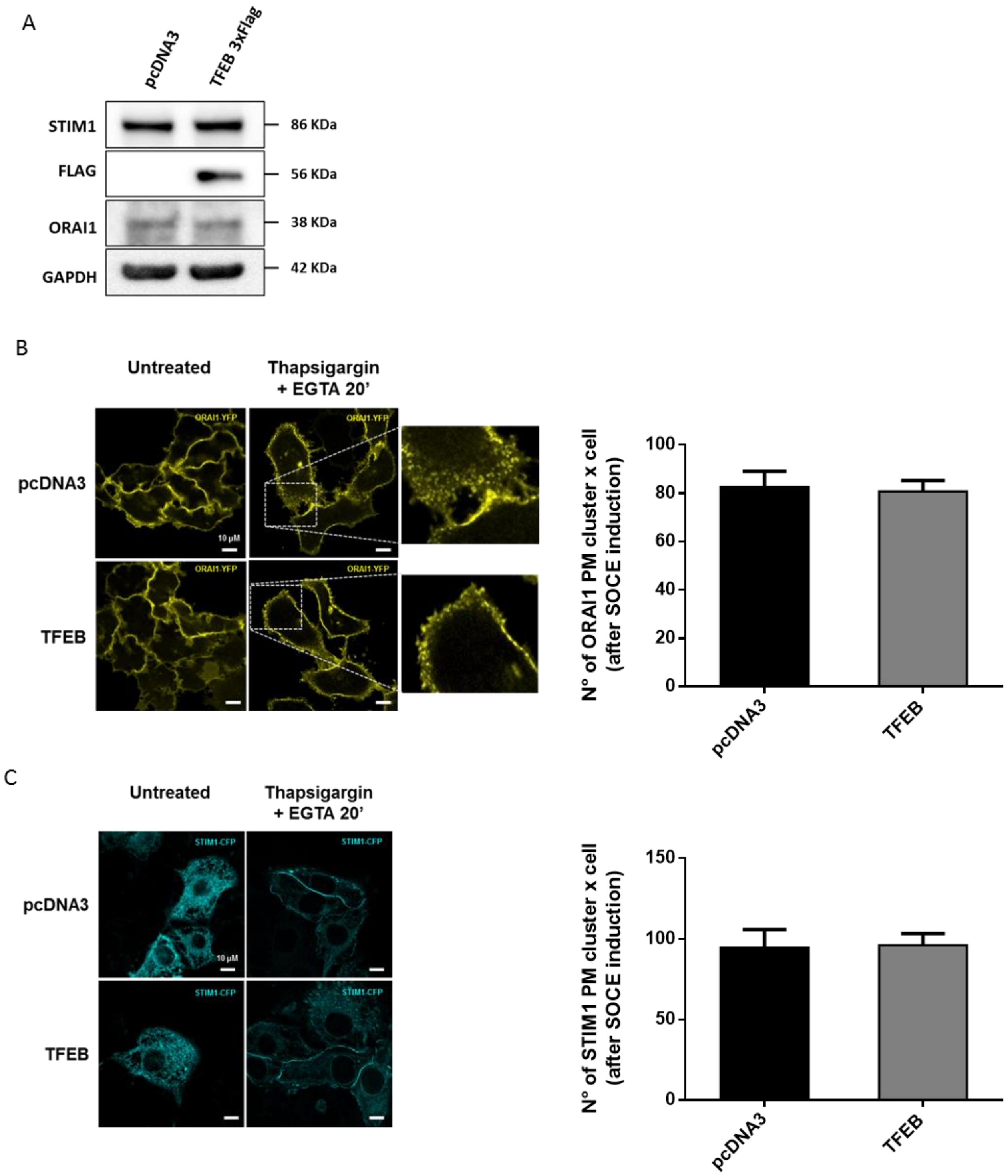

Supplementary Figure S7.

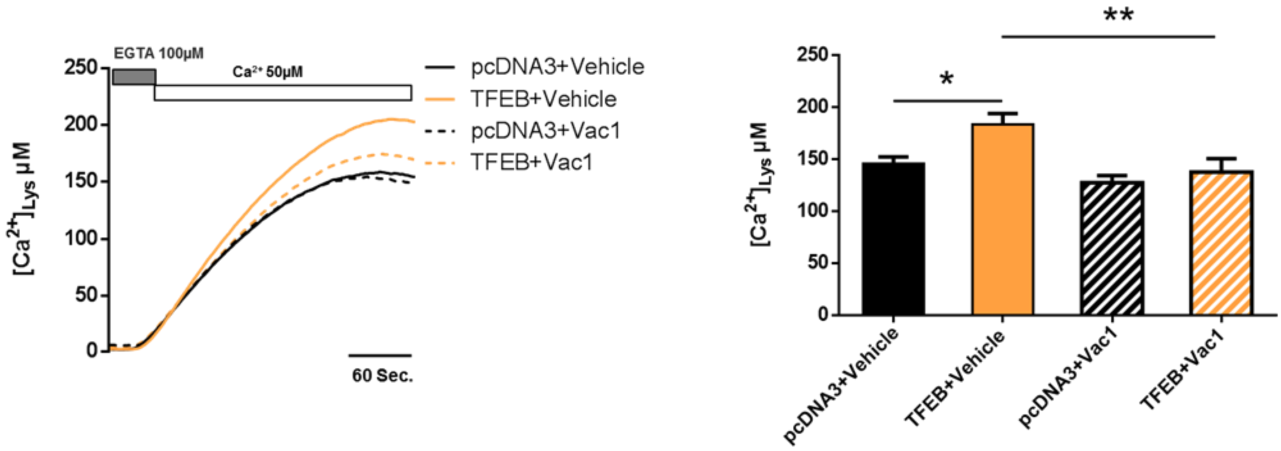

Supplementary Figure S8.

A

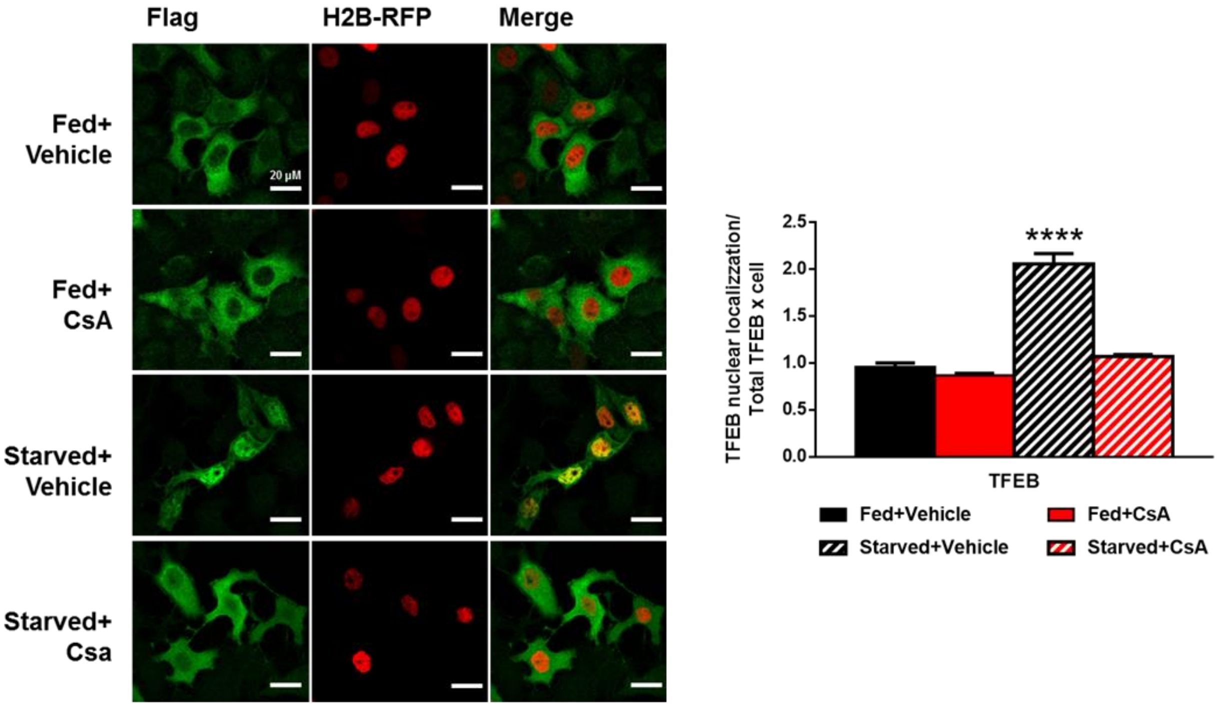

B

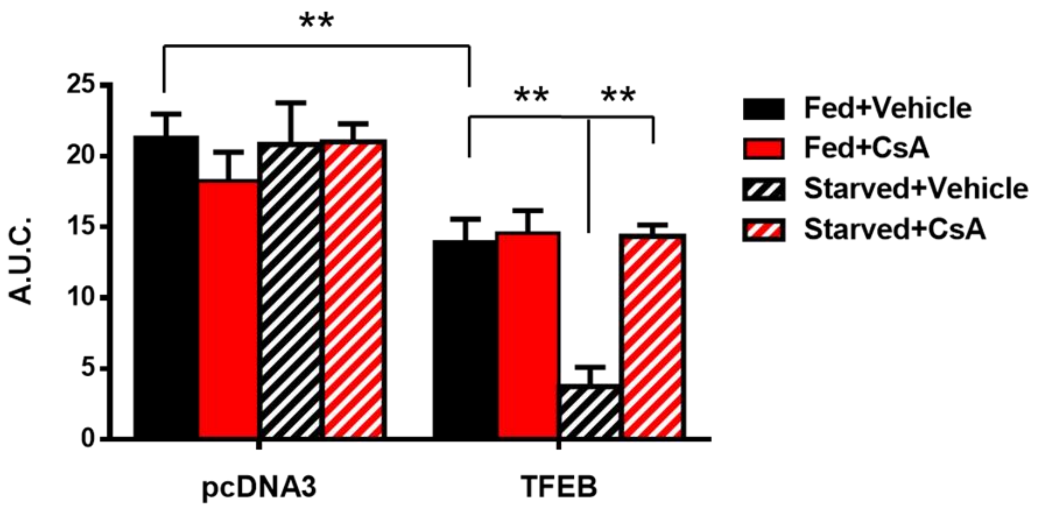

Supplement: Supplementary Information [file srep40797-s1.pdf]
